# Identification and characterization of *Tc1/mariner*-like DNA transposons in genomes of the pathogenic fungi of the *Paracoccidioides *species complex

**DOI:** 10.1186/1471-2164-11-130

**Published:** 2010-02-23

**Authors:** Marjorie M Marini, Tamiris Zanforlin, Patrícia C Santos, Roberto RM Barros, Anne CP Guerra, Rosana Puccia, Maria SS Felipe, Marcelo Brigido, Célia MA Soares, Jerônimo C Ruiz, José F Silveira, Patrícia S Cisalpino

**Affiliations:** 1 Departamento de Microbiologia, Instituto de Ciências Biológicas, Universidade Federal de Minas Gerais, 31270-901 Belo Horizonte, MG, Brazil; 2 Departamento de Microbiologia, Imunologia e Parasitologia, Escola Paulista de Medicina, Universidade Federal de São Paulo, 04023-062 São Paulo, SP, Brazil; 3 Laboratório de Biologia Molecular, Instituto de Ciências Biológicas, Universidade Federal de Goiás, 74001-970 Goiânia, GO, Brazil; 4 Laboratório de Biologia Molecular, Instituto de Ciências Biológicas, Universidade de Brasília, 70910-900 Brasília, DF, Brazil; 5 Centro de Pesquisas René Rachou, FIOCRUZ, 30190-002 Belo Horizonte, MG, Brazil

## Abstract

**Background:**

*Paracoccidioides brasiliensis *(Eukaryota, Fungi, Ascomycota) is a thermodimorphic fungus, the etiological agent of paracoccidioidomycosis, the most important systemic mycoses in Latin America. Three isolates corresponding to distinct phylogenetic lineages of the *Paracoccidioides *species complex had their genomes sequenced. In this study the identification and characterization of class II transposable elements in the genomes of these fungi was carried out.

**Results:**

A genomic survey for DNA transposons in the sequence assemblies of *Paracoccidioides*, a genus recently proposed to encompass species *P. brasiliensis *(harboring phylogenetic lineages S1, PS2, PS3) and *P. lutzii *(*Pb01-like *isolates), has been completed. Eight new *Tc1/mariner *families, referred to as Trem (**Tr**ansposable **e**lement **m**ariner), labeled A through H were identified. Elements from each family have 65-80% sequence similarity with other *Tc1/mariner *elements. They are flanked by 2-bp TA target site duplications and different termini. Encoded DDD-transposases, some of which have complete ORFs, indicated that they could be functionally active. The distribution of Trem elements varied between the genomic sequences characterized as belonging to *P. brasiliensis *(S1 and PS2) and *P. lutzii*. TremC and H elements would have been present in a hypothetical ancestor common to *P. brasiliensis *and *P. lutzii*, while TremA, B and F elements were either acquired by *P. brasiliensis *or lost by *P. lutzii *after speciation. Although TremD and TremE share about 70% similarity, they are specific to *P. brasiliensis *and *P. lutzii*, respectively. This suggests that these elements could either have been present in a hypothetical common ancestor and have evolved divergently after the split between *P. brasiliensis *and *P. Lutzii*, or have been independently acquired by horizontal transfer.

**Conclusions:**

New families of *Tc1/mariner *DNA transposons in the genomic assemblies of the *Paracoccidioides *species complex are described. Families were distinguished based on significant BLAST identities between transposases and/or TIRs. The expansion of Trem in a putative ancestor common to the species *P. brasiliensis *and *P. lutzii *would have given origin to TremC and TremH, while other elements could have been acquired or lost after speciation had occurred. The results may contribute to our understanding of the organization and architecture of genomes in the genus *Paracoccidioides*.

## Background

The thermodimorphic fungus *Paracoccidioides brasiliensis *is the etiological agent of paracoccidioidomycosis, a systemic endemic disease that affects at least 10 million people in Latin America [1]. Infection probably occurs as a result of inhalation of conidia that subsequently transform into yeast forms within the lung, resulting in asymptomatic infection or infection that can progress to acute (sub-acute) and chronic clinical forms of the disease [2]. Paracoccidioidomycosis is the 8th most common cause of death due to chronic/recurrent infections and parasitic diseases in Brazil [3].

The teleomorph (sexual) stage of this fungus is unknown, but analysis of the large rDNA subunit classified it as an Ascomycete, order Onygenales, family Onygenaceae, phylogenetically close to *Ajellomyces*, a genus that harbors 2 other human dimorphic fungal pathogens, specifically *Histoplasma capsulatum *and *Blastomyces dermatitidis *[4]. Molecular analysis by random amplification of polymorphic DNA (RAPD) [5], restriction fragment length polymorphism (RFLP) [6] and electrophoretic karyotyping showed extensive genetic variability among distinct isolates [7,8].

RAPD, a technique popular for its simplicity, has been useful in determining the existence of genetically distinct *P. brasiliensis *groups, their relationship with geographic distribution, and the similarity among clinical, animal and environmental samples. However, the information has been difficult to compare. Gene polymorphism studies were pioneered by characterization of Pb*GP43 *polymorphism, a gene encoding the fungus immunodominant antigen, in a sample of 17 isolates [9]. By comparing 2 sequenced PCR fragments from the whole gene (exon 1, intron and exon 2) these authors found 21 informative substitution sites, mostly in exon 2, which defined 5-6 genotypes. The maximum-likelihood phylogenetic tree generated with these sequences clearly reflected the presence of specific genetic groups in the species. Subsequently, multilocus genealogy studies demonstrated evolutionary lineages identifying the occurrence of cryptic phylogenetic species that were morphologically indistinguishable by following the criteria of genealogical concordance and non-discordance [10-12] which is used to identify the reduction of gene flow between groups of individuals due to geographic or reproductive isolation barriers. The Pb*GP43 *locus, whose characteristic genotypes have recently been reviewed [13], was the most polymorphic and informative in these studies. Matute et al. [10] analyzed the genetic structure of 65 *P. brasiliensis *isolates and concluded that they could be grouped into 3 distinct phylogenetic species: S1 (including isolate Pb18), PS2 (including isolate Pb03) and PS3 (composed exclusively of Colombian isolates). In a study of 21 *P. brasiliensis *isolates, 14 of which had been included in the above study, Carrero et al. [11] came to a similar conclusion for all the isolates, with the exception of isolate Pb01, which they suggested was a new phylogenetic species in the genus *Paracoccidioides*. Recently, Teixeira et al. [12], analyzing 88 isolates of the fungus, found that 17 of them were genotypically similar, belonging to the *Pb01-like *group. They estimated that the S1/PS2/PS3 species clade and the *Pb01-like *new species, for which the name *Paracoccidioides lutzii *was proposed, shared a common ancestor approximately 32 million years ago. These studies suggested that the genus *Paracoccidioides *consists of at least 4 different, previously unrecognized phylogenetic species: *P. brasiliensis *S1 (species), PS2 (phylogenetic species 2) and PS3 (phylogenetic species 3), and a 4th quite distinct lineage comprising fungal isolates formerly referred to as *Pb01-like *[11], now proposed to be a new species, *P. lutzii *[12]. The multilocus sequence analysis also supported recombination in nature in *P. brasiliensis *(S1) [10] and *P. lutzii *[11,12], indicating the presence of sexual reproduction. Thanks to The Broad Institute Fungal Genome Initiative (FGI) Project, the complete genome of representative isolates from *P. brasiliensis *S1 (Pb18), PS2 (Pb03) and *Pb01 like *(Pb01) have recently been made available at the site: http://www.broad.mit.edu/annotation/genome/paracoccidioides_brasiliensis thus allowing further and specific comparisons among isolates. Previous local projects, however, contributed to the construction of large expressed sequence tag databases of Pb18 [14] and Pb01 [15,16].

Transposable elements (TEs) represent a substantial fraction of eukaryotic genomes that have been found in virtually all species investigated to date. They are abundant in fungi and represent 3-20% of the genomes of many filamentous fungi [17,18]. TEs can produce a variety of effects in genomes, such as gene inactivation or modification, chromosome breakage, and genome recombination or rearrangement, thereby generating plasticity [19]. Karyotyping and gene mapping indicated the occurrence of chromosomal rearrangements [8], which are associated with the presence of repetition and TE in fungal genomes [17].

TEs can be grouped into 2 classes according to their transposition mechanism. Class I consists of retrotransposons that transpose via an RNA intermediate that is reverse-transcribed into DNA by a TE-encoded reverse transcriptase. Class II elements, also called DNA transposons, move via a DNA intermediate. Because of the abundance and diversity of TEs described in the eukaryotic genomes, a unified classification system for eukaryotic TEs has been proposed by different groups [20,21]. DNA transposons can be divided into 3 subclasses: the first comprises the elements that transpose via the classical "cut-and-paste" mechanism; the second comprises elements that transpose by replication involving the displacement of only one strand; and in the third, transposons move by a mechanism called "rolling-circle" [20,21].

Subclass 1 DNA transposons require the cleavage of both DNA strands for the transposition and have 2 orders - TIR and Crypton. Six superfamilies of the order TIR from subclass 1 have been identified in fungi: *Tc1/mariner, hAT, Mutator, Transib, PIF*-*Harbinger *and *CACTA*. TIR transposases contain a signature active site motif that consists of 3 acidic amino-acids (DDE or DDD) and forms the catalytic pocket of the enzyme responsible for cleaving DNA strands. Crypton, the only superfamily representative of the second order, has only been found to date in fungi and, unlike any previously described transposons, encodes a putative tyrosine recombinase instead of a classical transposase. This recombinase lacks an RT domain, which suggests that transposition occurs via a DNA intermediate. It does not contain long repeat sequences, but is bordered by short direct repeats that may have been generated by its insertion into the host genome by recombination. Cryptons may be excised from the host genome by tyrosine recombinase, generating a circular, extrachromosomal, double-stranded DNA molecule, for which cleavage of both DNA strands is required. Elements from subclasses 2 (Helitrons) and 3 (Maverick) have only one strand cleaved during transposition and have also been identified in fungi, but the *Tc1/mariner *superfamily is probably the most prevalent in these organisms [20-22].

Transposons contain terminal inverted repeats (TIRs), which are recognized by the pipsqueak helix-turn-helix (HTH) motif in the transposase domain. Each subclass 1 DNA transposon superfamily is characterized by a specific transposase core that is different from those of other superfamilies. Upon insertion, DNA transposons generate target site duplications (TSD) with lengths that are relatively well conserved among superfamily members [20,21].

TEs have been poorly characterized in *P. brasiliensis*. Transcriptome analysis indicated that Ty-like retroelements were among the most abundant ESTs in *P. brasiliensis *[15]. Nascimento *et al*. [23], searching for microsatellites that could be potentially useful as molecular markers, reported that a small number of them were located in transposons. The focus of our work has been the characterization of DNA transposons and their derivatives in the genus *Paracoccidioides*. To this end we searched for DNA transposons in the *Paracoccidioides *genomic sequence database http://www.broad.mit.edu/annotation/genome/paracoccidioides_brasiliensis. We have discovered 7 new families of DNA transposons and one new subfamily of Mariner-1_AF belonging to the *Tc1/mariner *superfamily. Remarkably, some Trem (Transposable element mariner) elements appear that may be active in the genomes of representative isolates of 2 different *Paracoccidioides *phylogenetic lineages. The finding of active autonomous DNA transposons in *P. brasiliensis *may have implications for an understanding of the evolutionary processes underlying the diversification of this group. Furthermore, transposons are efficient vectors for introducing foreign DNA into cells.

## Results

### Occurrence of DNA transposons in the genus *Paracoccidioides*

A survey of the *P. brasiliensis *Pb01 Functional and Differential Genome Project annotated EST database (PbDBEST) [24] for sequences similar to transposase sequences found 2 contigs (530 and 1938, with 673 and 637 bp respectively) that contain open reading frames (ORF) encoding proteins of 155 and 71 amino acids, respectively, with similarity to the conserved HTH DNA binding domain of the transposase of the *Aft*1 DNA transposon in *Aspergillus fumigatus*. These sequences were amplified from *P. brasiliensis *genomic DNA, their identities confirmed by sequence analysis, and then used as probes in Southern blot hybridization with genomic DNA of different *P. brasiliensis *isolates. This revealed the presence of multiple copies of DNA transposons dispersed throughout the genome of different isolates. RT-PCR analysis of total RNA of the isolates Pb01 (recently proposed to be a new species, *P*. *lutzii*) [12], Pb03 and Pb18 (*P. brasiliensis*, corresponding to distinct phylogenetic lineages known, respectively, as S1 and PS2) [10] confirmed transcription of the putative elements (data not shown).

The transposon sequence from each EST contig was used as a query in a modified BLASTN search (see Methods section for details) against the whole genome sequence assemblies of all 3 *Paracoccidioides *genomes (Pb01, Pb03 and Pb18) and combined with the strategy for identification of TIRs, what allowed the Artemis annotation tool to be used to identify transposons.

Table 1 shows the number of sequence copies with similarity to transposases identified in the genome of each isolate sequenced. In total, 1332 hits were unambiguously identified in the genomes of Pb01 (n = 384), Pb03 (n = 475) and Pb18 (n = 473). They were predicted to have features that allowed them to be classified in the *Tc1/mariner *superfamily. A significant number of the hits found (n = 868; 65.2%) corresponded to highly defective elements, with lower conservation of the typical transposase domains (HTH, CENPB and/or DDE), which have still to be analyzed for classification (Table 1).

**Table 1:** Abundance of DNA transposons identified and classified in the different isolates of *P. brasiliensis*^a^

	Pb01	Pb03	Pb18	Total
**Classified^b^**	102 (26.6%)	177 (37.3%)	185 (39.1%)	464 (34.8%)
**Not classified^c^**	282 (73.4%)	298 (62.7%)	288 (60.9%)	868 (65.2%)
**Total**	384 (100%)	475 (100%)	473 (100%)	1332 (100%)

^a^DNA transposase sequences from the *P. brasiliensis *transcriptome were used to perform queries in a modified BLASTN search (see Methods) in the genome of isolates Pb01, Pb03 and Pb18 and combined with a strategy for identification of TIRs.

^b^Elements assigned to a DNA transposon superfamily according to the standard principles of the TE classification proposed by Wicker et al. [20].

^c^Sequences that do not meet all the criteria proposed by Wicker et al. [20]. They are highly defective elements, with lower conservation of the typical transposase domains (HTH, CENPB and/or DDE).

We also searched the *Paracoccidioides *genomes for *Cryptons*, rolling-circle DNA transposons (Helitrons) and self-synthesizing DNA transposons (Polintons) [20,21], using consensus sequences for these elements (tyrosine recombinase, helicase, replication protein A and DNA polymerase B), no significant matches were found.

In the genomic sequence of Pb01 (*P. lutzii*), only 3 DNA transposon families have been classified, corresponding to 102 (26.6%) of the 384 hits found in this isolate (Table 1). In the set of elements for Pb03, 6 full-length potentially functional autonomous elements coding for putative transposases with well-defined TIRs were classified. The copies of the 6 elements found in Pb03 totaled 177 hits, or 37.3% of the 475 hits, similar to DNA transposons in this isolate (Table 1). Of the 473 hits registered in Pb18, 185 (39.1%) were identified and correlated with *Tc1/mariner *elements.

### *Tc1/mariner *superfamily in the genus *Paracoccidioides*

**Figure 1:**
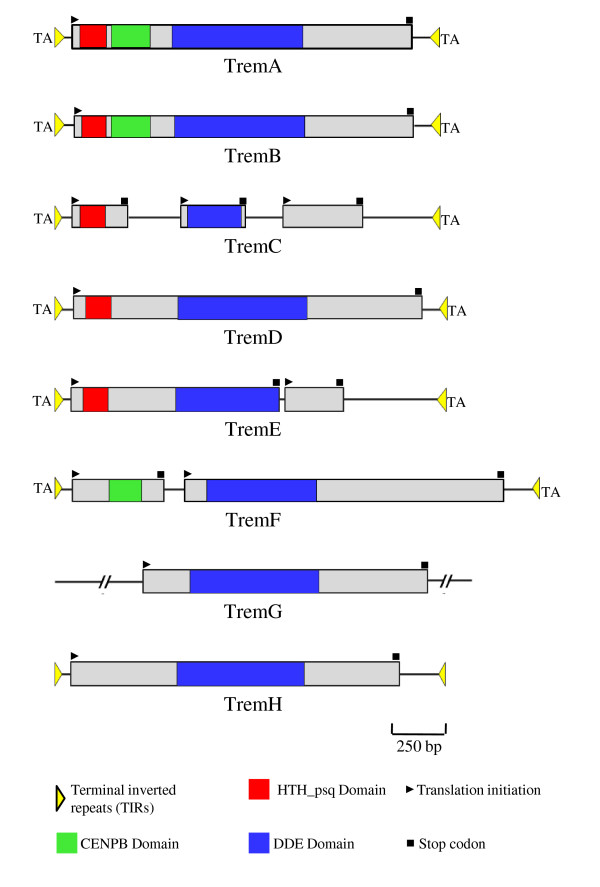
**Structure and organization of *Tc1/mariner *like elements in *P. brasiliensis***. Reconstruction of consensus sequences from Trem elements identified in 3 isolates of *P. brasiliensis*. The boxes represent the ORFs in which the translation initiation and stop codons are represented by the black triangle and black square, respectively. The red boxes represent the Helix-Turn-Helix_pipsqueak (HTH_psq) conserved domain, green boxes the Centromere binding domain (CENBP), blue boxes the DDE conserved domain, and yellow triangles the terminal inverted repeats (TIRs).

**Figure 2:**
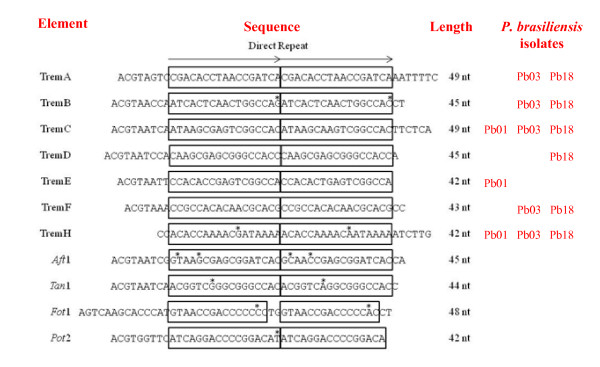
**Terminal Inverted Repeats (TIRs) of Trem elements**. Structure of TIRs of Trem elements and comparison with TIRs from other fungal *Tc1/*mariner transposons (*Aft*1 from *Aspergillus fumigatus*, *Tan*1 from *Aspergillus nidulans*, *Pot*2 from *Magnaporthe grisea *and *Fot*1 from *Fusarium oxysporum*). The boxes are the internal direct repeats, and the asterisks indicate the mismatches.

We identified 8 DNA transposon elements in *Paracoccidioides *that share 65-80% identity at the nucleotide level with *Tc1/mariner *transposons deposited in Repbase [25] (details in Additional file 1) at the Genetic Information Research Institute (GIRI). Like the *Tc1/mariner *transposons, 6 of these elements are flanked by TA dinucleotide (Figure 1). Because of their similarity to the *Tc1/mariner *superfamily and the fact that most of them (61.6%) insert specifically in TA target sites, these elements were given the name Trem (an abbreviation for Transposable element mariner). The completeness of transposons was confirmed by the presence of TIRs at both ends, followed by multiple sequence alignment to well-studied examples (Table 2, Figure 2). Incomplete sequences were not included in the comparative analyses shown in Figure 2. We examined 464 insertion loci of Trem elements (Table 2), of which 321 (69.2%) are flanked by well-defined TIRs, while the remaining sequences, including all 17 TremG elements, are not flanked by TIRs.

**Table 2:** Characteristics of the *Tc1/mariner *transposons identified in the genome of *P. brasiliensis*.

Transposon	TSD^a^	Repeat Masking^b^	Size^c ^(bp)	Protein^d ^(aa)	TIRs^e ^(bp)	Copy number^f^			
						
						Pb01	Pb03	Pb18	N^g^
TremA	TA	66% (Mariner-1_AF)	1850	547	49	-	7 (5)	11(5)	18
TremB	TA	65% (Mariner-1_AF)	1857	544	45	-	15 (2)	21	36
TremC	TA	66% (Mariner-2_AO)	1869	95, 103, 127	49	48	67	50	165
TremD	TA	80% (Mariner-1_AF)	1891	559	45	-	-	5 (4)	5
TremE	TA	65% (Mariner-1_AF)	1883	335, 217	42	25	-	-	25
TremF	TA	67% (Mariner-9_An)	2329	143, 505	43	-	12	25	37
TremG	-	66% (Mariner-5_AF)	-	460	-	-	6	11	17
TremH	-	66% (Mariner-3_AO)	1882	525	42	29	70	62	161
Total						102	177	185	464

^a^TSD - Target Site Duplication. TA dinucleotide.

^b^Repeat Masking - results of the analysis performed with the Repeat Masking script using the Girinst http://www.girinst.org/repbase database and percentage similarity.

^c^Size - total length of element in base pairs (bp)

^d^Protein - Length of transposase in amino acids (aa). Some elements contain 2 or 3 ORFs encoding different proteins (see Figure 1).

^e^TIRs - Length of Terminal Inverted Repeats in base pairs (bp).

^f^Copy number - Number of TE copies found in the genome of the 3 *P. brasiliensis *isolates. The number of elements with potentially functional ORFs is shown in parentheses.

^g^N - The total number of *Tc1/mariner *transposons identified in *P. brasiliensis*.

There are 7 copies of TremA in the Pb03 isolate, 5 which contain a full-length ORF and 2 which are truncated, and 11 copies in the Pb18 isolate, 5 which have an intact ORF (Figure 1, Table 2). TremA sequences were aligned to build an 1850 bp consensus containing a single ORF (nt 85-1728) that encodes a 547 amino acid protein with 3 conserved domains characteristic of transposases - HTH, CENPB and DDE (Figure 1). The TremA TIRs are 49 bp long and have a 17 bp internal direct repeat (Figure 2). Analysis of TremA with the RepeatMasker script in the Girinst database http://www.girinst.org/repbase revealed a similarity of only 66% with the *A. fumigatus *Mariner-1_AF transposon, suggesting that it may be a new DNA transposon family [20].

We found 36 copies of TremB, 15 in the genome of the Pb03 isolate and 21 in Pb18 (Table 2). However, only 2 copies showed intact ORFs, which were used to generate a 1857 bp consensus sequence flanked by 45 bp TIRs (Table 2, Figures 1 and 2). This ORF (nt 130-1764) encodes a 544 aa transposase with 3 conserved domains (HTH, CENPB and DDE; Figure 1). Analysis using RepeatMasker gave an index of 65% similarity with the Mariner-1_AF. It is interesting that 25 TremB sequences carrying truncated ORFs are flanked by intact TIRs.

TremC is the most abundant DNA transposon, having 165 truncated copies dispersed throughout the genomes. The 32 longest sequences were aligned to generate a 1869 bp consensus flanked by 49 bp TIRs (Table 2, Figures 1 and 2). It contains 3 short ORFs, 2 which encode the HTH and DDE domains (Figure 1). The central region of the element shows similarity with *A. fumigatus *transposases, and analysis by RepeatMasker revealed 66% similarity with Mariner-2_AO in *Aspergillus oryzae*. The TremC shown in Figure 1 is based on the element's most conserved sequences.

TremD is present in a small number of copies (n = 5), exclusively in the Pb18 isolate (Table 2). The consensus sequence has 1891 bp and harbors a 1680 bp ORF (nt 95-1774) flanked by 45 bp TIRs (Figures 1 and 2). The ORF codes for a 559 aa transposase carrying the HTH and DDE domains (Figure 1). RepeatMasker analysis showed 68% similarity with Mariner-1_AF in the central region, and 80% similarity with the TIRs, suggesting that TremD is related to or belongs to this family [20].

The consensus sequence for the TremE transposon, found exclusively in the Pb01 isolate, is 1883 bp long, flanked by 42 bp TIRs (Table 2, Figures 1 and 2). It includes 2 ORFs: one has 1008 bp (nt 78-1085) and codes for a 335 aa transposase with the HTH and DDE domains; and the second (654 bp long, nt 1113-1766) encodes a 217 aa protein similar to a hypothetical protein (1e-47) from *Ajellomyces capsulatum *(Figure 1). TremE showed a 65% similarity with the Mariner-1_AF transposon.

With its 2329 bp consensus sequence, TremF is the longest of the elements described in this study and the third most abundant (n = 37) (Table 2, Figure 1). The consensus sequence is flanked by 43 bp TIRs and contains 2 ORFs: the first is 432 bp long (nt 89-520) and codes for a 143 aa protein harboring the conserved domain CENPB; the second extends from position nt 650 to 2167 (1518 bp) and codes for a 505 aa DDE-domain-harboring protein (Figures 1 and 2). The results of RepeatMasker analysis revealed a 67% similarity with Mariner-9_AN in *Emericella nidulans*, which is a non-autonomous DNA transposon belonging to the *pogo *clade. Thus, TremF could represent a new transposon family [20].

TremG, the 7th *Tc1/mariner *element we identified, has 6 copies in Pb03 and 11 in Pb18 (Figure 1, Table 2). All copies are truncated, flanked by highly divergent and irregular sequences (35-124 bp). The central core of some elements has an ORF coding for a 460 aa protein carrying the DDE domain. Unfortunately, it was not possible to determine the precise boundary of this element because of the divergence among the flanking sequences. RepeatMasker analysis showed a 66% similarity with the Mariner-5_AF in *A .fumigatus*, a DNA transposon of the *Tc1 *clade. For this reason, this element was classified as a *Tc1/mariner *transposon.

TremH is present in the 3 isolates, with 29 copies in Pb01, 70 in Pb03 and 62 in Pb18 (Figure 1, Table 2). Of the 161 copies of this element, 120 (74.5%) are flanked by 42 bp TIRs; however, it was not possible to define its target site. We used 6 copies that had the best sequence conservation (94% identity among them) and encoded the DDE motif to rebuild the 1882 bp consensus sequence (including two 42 bp flanking TIRs). RepeatMasker analysis showed a 66% similarity with the Mariner3_AO in *A .oryzae*. The element copy contains an ORF coding for a 525 aa protein carrying the DDE motif (Figure 1), similar to that in the transposon in *Talaromyces stipitatus *(2e-102) (Table 2). In this region, 3 strictly conserved acidic amino acids - D(176), D(289) and D(325) (positions relative to the transposase sequence) - were found, and these probably constitute the DDE catalytic motif in TremH. Since the transposase activity in many DNA transposons relies upon the catalytic activity of the DDE domain, we suggest that TremH is a *Tc1/mariner*-like transposon.

### Phylogenetic analysis

**Figure 3:**
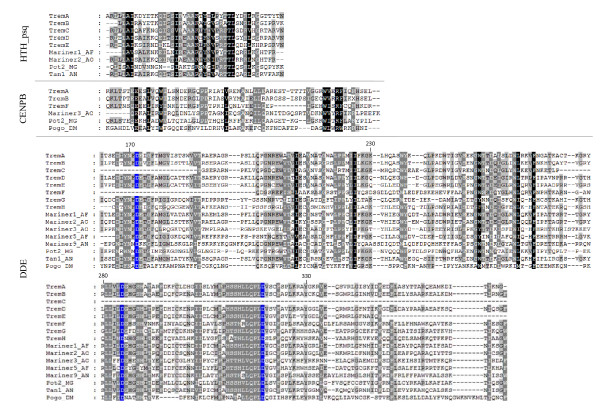
**Comparison of transposase domains of Trem elements and related fungal transposases**. ClustalW alignment of the conserved domains HTH_psq, CENPB and DDE of Trem elements with the domains of the fungal transposable elements Mariner1_AF and Mariner5_AF from *Aspergillus fumigatus*, Mariner2_AO, Mariner3_AO from *Aspergillus oryzae*, Mariner9_AN from *Aspergillus nidulans, Tan1 *from *Aspergillus niger *and *Pot*2 from *Magnaporthe grisea*. The *Pogo *element from *Drosophila *is also included to illustrate the conservation between kingdoms. Sequences corresponding to the conserved residues are shaded in black (100% conservation), dark ash (80% conservation) and ash (60% conservation). The DDD motif, characteristic of the catalytic domain of transposases, is highlighted in blue. Numbers indicate distance along TremA.

The alignment of the predicted sequences corresponding to each of the conserved transposase domains found in 8 DNA transposons in *Paracoccidioides*, with the sequences of well-characterized DNA transposons of the *Tc1/mariner *superfamily (Figure 3) from other species, allows the identification of motifs considered essential for active transposition (Figure 3). Some of the *Paracoccidioides *transposases are interrupted by stop codons, minor indels or the absence of a translation initiation codon (Table 2, Figure 1 and Additional file 2). The complete catalytic triad DDE occurred in 6 of the elements (TremA, B, D, E, G and H) and was characterized by the substitution of the glutamate residue by aspartate to form a D, D(35 residues), D motif, as previously observed in other fungal transposases. The triad signatures (the triad residues and the distances between them) are very similar and can be represented by D10-(111 residues)-D122-(35 residues)-D158 in the elements TremA, B, D and E; D10-(109 residues)-D120-(35 residues)-D156 in the element TremG; and D7-(111 residues)-D119-(35 residues)-D155 in the element TremH. In TremF, the first aspartic residue was deleted, but the distance between the second and third aspartate residues was maintained (-D89-(35 residues)-D125). In TremC, all 3 aspartic residues were absent.

In addition to the conserved catalytic triad DDD, transposases contain a number of other highly conserved, group-specific amino acids, such as the HTH and CENPB domains. The HTH domain was found in 5 Trem elements (TremA, B, C, D and E) with varying degrees of conservation - 58% identity between the pairs TremA/TremB, TremB/TremC and TremD/TremE, and 53% identity between TremA/TremC. The identities between TremE/TremA and TremE/TremB were lower than 40%. Alignment of the DNA-binding domains of the transposases revealed a distinction between Trem elements, which may be functionally connected to the TIRs of these elements.

When the HTH domain was compared with that of other fungal transposases, the highest identity index (60%) was found between TremD and the *Tan*1 transposon of *Aspergillus niger*. The CENPB domain was present in 3 of the *Paracoccidioides *transposons (TremA, B and F). The identity between TremA and TremB was 65.6%, and between these elements and Trem F less than 36%.

It was also observed that 3 elements (TremA, TremB and TremD) presented conserved Cx2Cx4Hx4C zinc finger domains in the C-terminal ends of their transposases corresponding, respectively, to amino acid residues 531-544, 528-541 and 540-553. In TremC and TremE, similar but non-canonical motifs were present at the C-terminal regions corresponding, respectively, to the residues 290-315 (CCx18Cx4H) and residues 404-419 (Cx2Cx2Hx8G).

**Figure 4:**
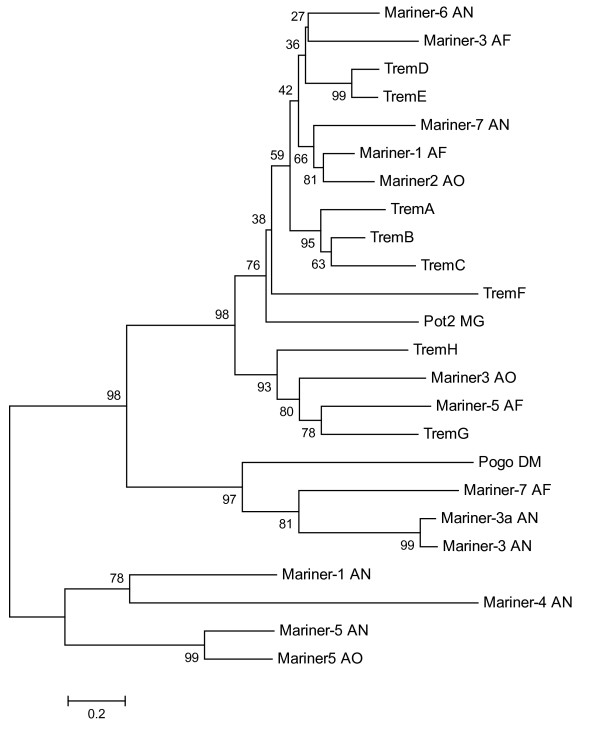
**Phylogeny of Trem elements and related fungal transposons**. Phylogenetic tree of the predicted amino acid sequences of Trem elements of *P. brasiliensis *and different *Tc1/mariner *transposons. The list of the sequences used to construct alignments and phylogenetic trees is given in the Methods section. Neighbor-joining trees were constructed using the equal-input model with 5,000 bootstrap replicates. Numbers near the individual nodes denote BioNJ bootstrap values. The scale bar corresponds to 0.2 substitutions per site.

Phylogenetic analysis using amino acid sequences clustered the transposases of *Paracoccidioides *into 2 major groups, one carrying TremA, B, C, D, E and F elements, and the other harboring TremH and G elements (Figure 4). This analysis confirms the proximity between TremA, TremB and TremC elements (95% bootstrap) and TremD and TremE elements (99% bootstrap). TremF elements, although clustered with the Trem elements above, are divergent and located in a distinct branch. Based on phylogenetic reconstructions (Figure 4), TremG and TremH seem to form a cluster completely separated from other Trem elements. Both groups contain *Tc1/mariner *transposases that are also present in other fungi, e.g. *A. nidulans, A. fumigatus *and *A. oryzae*.

Our analysis suggests that Trem elements of the different *Paracoccidioides *genomes were derived from a single ancestral sequence present before they diverged from one another. At a later point in the evolution of this fungus, transposases split into 2 separate lineages, giving rise to the family composed of TremA, B, C, D, E and F and the other consisting of TremH and G.

### Distribution and genomic environment of Trem elements

To characterize the insertion target sites and preferences in *Paracoccidioides *genomes in more detail, junction fragments between the transposon and the genome were analyzed. Trem elements, like other *Tc1/mariner *transposons, integrate into a TA dinucleotide, causing target-site duplication of the TA sequence at the TIR boundary. Additional file 2 lists 321 independent insertion events identified in the 3 *Paracoccidioides *genomes, demonstrating integration of the transposon into a TA dinucleotide within the fungal genome and validating genuine transposition.

Analysis of the genomic location of each Trem element insertion showed that they were dispersed across the genomes. They did not, however, appear to be completely random (additional file 3). Genome sequences from isolate Pb03 (29.05 Mb) were assembled in 65 supercontigs (scaffolds). The 475 hits similar to DNA transposons (Table 1) were found along 53 of the 65 supercontigs of Pb03. Of these 53, 39 contain Trem elements (n = 177), which account for 1.14% of the genome of isolate Pb03 (additional file 4). In the genome of Pb18 (29.95 Mb), there are 473 hits similar to DNA transposons (Table 1) distributed in 44 of the 57 supercontigs, and Trem elements (n = 185) were found in 36 supercontigs of this isolate (additional file 5), covering 1.11% of the genome. In isolate Pb01 (genome size 32.94 Mb, 111 supercontigs), the 384 hits showing similarity to TEs (Table 1) were distributed along 87 supercontigs (approximately one element each 85 kb), and Trem elements accounted for 0.58% of the genome (additional file 6).

**Figure 5:**
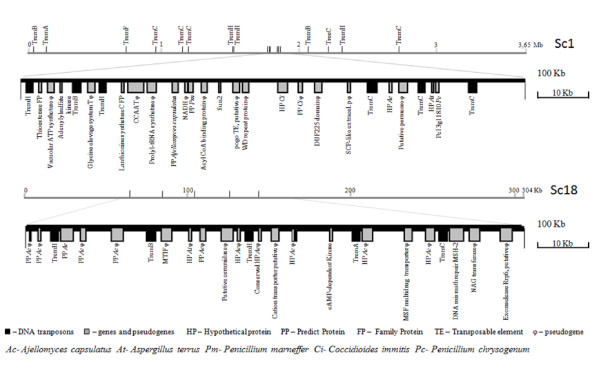
**Genomic environment of Trem elements**. Schematic representation of 2 *P. brasiliensis *supercontigs (Sc1 and Sc18) harboring Trem elements; each line represents the entire contig. The length is indicated to the right of each contig. The position of Trem elements is indicated. The dotted rectangle delineates the zoom-in area for each contig. The areas shown are 100 kb in length, and the exact position in the contigs is indicated below each line. Locus names appearing below genes correspond to their systematic names assigned in GenBank. The symbol φ indicates a pseudogene. The contigs have the following NCBI accession numbers: Sc1 - DS544803, Sc18 - DS544820.

The above results suggest that Trem elements do not integrate in a completely random fashion. In an extended survey, the genomic context in which Trem elements were inserted was examined in 100 kb region of supercontigs 1 (length 3.65 Mb) and 18 (length 304 kb) of isolate Pb03 (Figure 5). In supercontig 1, this particular region has a high density of DNA transposons (7 hits), 6 of which belong to the Trem family (3 TremC, 2 TremH and 1 TremB) (Figure 5). In addition to the 6 Trem elements, 10 genes (including those coding for thioesterase, lanthionine synthetase and hypothetical proteins) and 12 pseudogenes are present. Among the pseudogenes there is an unclassified *pogo*-like putative DNA transposon. In supercontig 18, we found 5 Trem elements (2 TremH, 1 TremA, 1 TremB and 1 TremC), 3 genes (cAMP kinase dependent protein, MSH2 and hypothetical protein) and 17 pseudogenes. The analysis showed that Trem elements target a wide variety of chromosomal positions and some appearing to be clustered in several regions of the genome.

### Southern blot and chromoblot hybridization analyses

**Figure 6:**
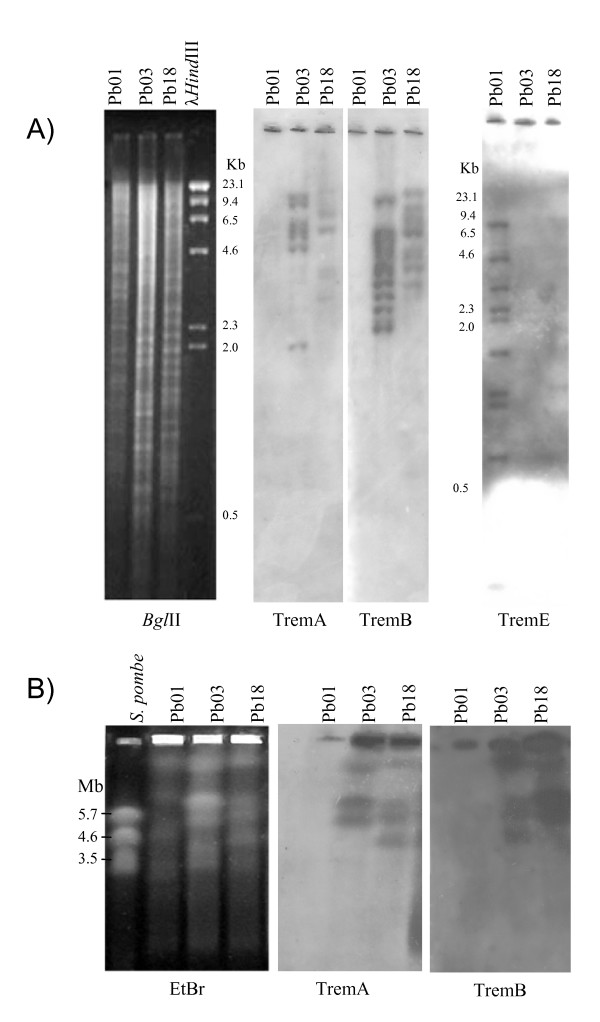
**Genomic Southern blotting analysis and chromosomal distribution of TremA and TremB elements**. A) Genomic DNA of isolates Pb01, Pb03 and Pb18 was digested with *Bgl*II, blotted onto nylon membranes and hybridized with TremA, TremB and TremE probes derived from the internal region of each element. B) Chromosomal distribution of TremA and B elements in different *P. brasiliensis *isolates. On the left are the molecular karyotypes of *P. brasiliensis *isolates Pb01, Pb03 and Pb18. Chromosomal bands were separated by pulsed-field gel electrophoresis and stained with EtBr. The autoradiograms from Southern hybridization using the elements TremA and TremB as probes are shown on the right.

Southern blot hybridization of *Paracoccidioides *genomic DNA with TremA, TremB and TremE probes revealed multiple bands, suggesting that multiple copies were inserted with polymorphic distribution (Figure 6A). Differences between the hybridization profiles obtained with the same Trem probe confirm the existence of different copy numbers and distribution of the element in the isolates. No hybridization signal was observed in isolate Pb01 (*P. lutzii*) with probes TremA or TremB. TremE probe hybridized with 10 well-defined bands in Pb01 isolate, and weakly with a 1.0 kb band in Pb18 isolate (*P. brasiliensis; *Figure 6A). Probe TremA hybridized with 3 of the 4 chromosomal bands observed in Pb03 and with 4 of the 5 in Pb18. Chromoblot hybridization also confirmed the absence of TremA and TremB in isolate Pb01 (Figure 6B). The *P. brasiliensis *synteny map shows the location of 17 scaffolds in the 5 chromosomes of isolate Pb18: http://www.broadinstitute.org/annotation/genome/paracoccidioides_brasiliensis/Chromomap.html. TremA and TremB elements are present in 4 chromosomes, but are not found in chromosome 5, in accordance with their karyotype profiles (Figure 6B).

### PCR amplification of Trem elements in *Paracoccidioides *isolates of distinct phylogenetic origin

TIRs of Trem elements consist of two 17 bp direct repeats similar to those found in DNA transposons of other fungi, namely *Aft*1 and *Tan*1 (Figure 2). The repeats are completely conserved except for the TIRs of the elements TremB and TremH, in which one substitution was detected (Figure 2). PCR screening using primers derived from TIRs of each Trem element was performed on genomic DNA from 17 clinical isolates, including those from the FGI genome project, and one environmental isolate (Table 3).

**Table 3:** *Paracoccidioides *isolates used in this study.

Isolate	Origin	Country	Phylogenetic Species^c^
Pb01^**a e f**^	clinical	Brazil - Goiás	*Pb01-like*
Ed01^**g**^	clinical	Brazil - Goiás	*Pb01-like*
1578^**g**^	clinical	Brazil - Goiás	*Pb01-like*
Pb03^**a d f h i**^	chronic PCM^b^	Brazil - São Paulo	PS2
Pb4^**d e f h i**^	chronic PCM	Brazil - São Paulo	PS2
Pb2^**d e f h i**^	chronic PCM	Venezuela	PS2
Pb18^**a d e f h i**^	chronic PCM	Brazil - São Paulo	S1
B339^**d e f h i**^	chronic PCM	Brazil - São Paulo	S1
Pb6^**d f h i**^	chronic PCM	Brazil - Paraná	S1
Pb5^**h i**^	chronic PCM	Brazil - Paraná	S1
Utero^**g**^	chronic PCM	Argentina	S1
Pb9^**d f h i**^	chronic PCM	Brazil - São Paulo	S1
Pb11^**d f h i**^	acute PCM	Brazil - Paraná	S1
Pb13^**d f h i**^	acute PCM	Brazil - Goiás	S1
Pb10^**d f h i**^	acute PCM	Peru	S1
Pb14^**d f h i**^	acute PCM	Brazil - São Paulo	S1
Pb12^**e h i**^	acute PCM	Argentina	S1
Penguin^**d f**^	penguin faeces	Uruguay	S1

^**a**^*P. brasiliensis *isolates used in the genome sequencing project - Broad Institute MIT and Harvard.

^**b**^PCM - paracoccidioidomycosis

^**c**^Phylogenetic Species grouped in accordance with Matute et al. [10]^**d**^, Carrero et al. [11]^**e**^, Teixeira et al. [12]^**f **^and Figure 7 (this work)^**g**^.

^**h**^Isolate was also used by Morais et al. [9]

^**i**^Revised by Puccia et al. [13].

**Figure 7:**
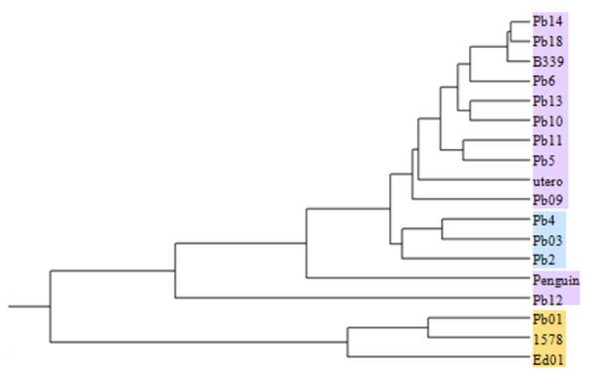
**Dendrogram of random amplified polymorphic DNA (RAPD) analysis of 18 *P. brasiliensis *isolates**. Dendrogram built from the results of RAPD analysis of genomic DNA of 18 *P. brasiliensis *isolates using 6 random primers (see Methods). The RAPD analysis allowed clear differentiation of the 18 isolates into 3 distinct groups *Pb01-like*, S1 and PS2. The *Pb01-like *isolates are highlighted in salmon, the S1 isolates in lilac and the PS2 isolates in light blue

Of the 18 isolates, 15 had been previously genotyped by sequencing and their phylogenetic relatedness was known; the phylogenetic relatedness of the remaining 3 isolates - 2 Brazilian (Ed01, 1578) and one Argentinean (utero) which had not been included in previous sequencing and multi-locus studies - was unknown (Table 3). Previous work using RAPD for typing *P. brasiliensis *indicated that the employment of certain primers and conditions allowed discrimination of isolates with very low genetic similarity and clustering of the most similar ones [5,6,26,27]. We considered that performing RAPD with these highly discriminatory primers would allow us to build a dendrogram in which the genetically most distant branches would correspond to the species level, *P. brasiliensis *and *P. lutzii *(Pb01-like), clustering isolates corresponding to the phylogenetic species S1 and PS2, an assumption that would uncover the phylogenetic relatedness of the 3 unknown isolates (Ed01, 1578 and utero). The 18 isolates submitted to RAPD analysis were classified as belonging to 3 of the 4 known phylogenetic lineages: S1 and PS2 (*P. brasiliensis*) or Pb01-like (*P.lutzii*) [10-12] (Figure 7). Analysis of the dendrogram showed 2 major clusters with a low coefficient of similarity (approximately 6%), the first corresponding to *P. lutzii *(Pb01, Ed01 and 1578). The second branch comprised all 15 *P. brasiliensis *isolates showing 2 internal clusters (80% similarity), the first including 10 S1 and the second clustering 3 PS2 isolates (Pb2, Pb03 and Pb4; Table 3) [10-12]. The *P. brasiliensis *isolates Penguin and Pb12, although previously defined as S1 (Table 3), did not group with the S1 isolates but appear as 2 branches each with only one strain. It is interesting that the isolates Penguin and Pb12 are from Uruguay and Argentina, while the majority of the other isolates are Brazilian (13, one Venezuelan - Pb2- and one Peruvian - Pb10). A fungal associated feature that has been reported to correlate by RAPD branching or clustering of *Paracoccidioides *isolates refers to the isolate's geographic origin [6].

**Figure 8:**
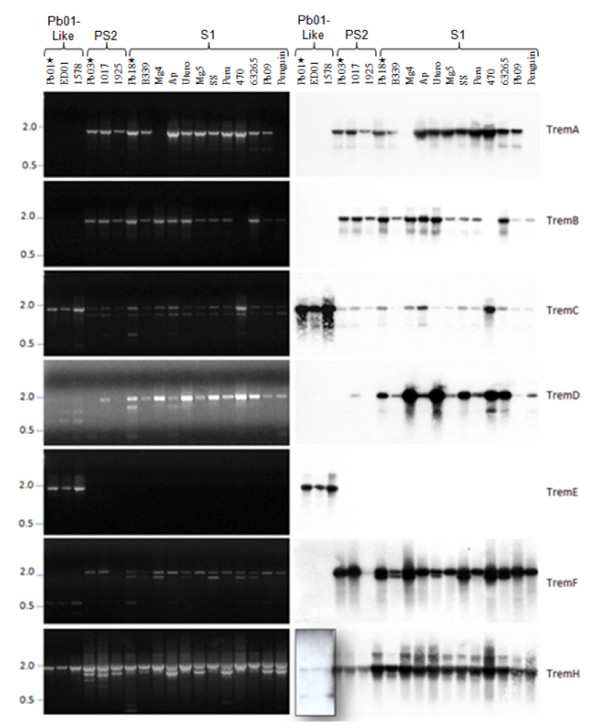
**PCR screening for Trem elements in different phylogenetic lineages of *P. brasiliensis***. Left: Electropherogram results from PCR analysis of a phylogenetically diverse panel of *P. brasiliensis *isolates. Oligonucleotide primer pairs are given in Additional File 7. PCR amplification of Trem elements in the genomic DNA of 18 isolates of *P. brasiliensis*, including cryptic species, using primers from the TIRs of each element indicated. Asterisks (*) indicate the *P. brasiliensis *isolates sequenced in the FGI of Broad Institute. Right: PCR amplicons were blotted onto nylon membranes and hybridized with Trem probes. Because the copy-number of each element differs among the *P. brasiliensis *isolates, exposure times for autoradiograms were not equivalent. They were: 20 h for TremD; 1 h for TremC and TremF; 10 min for TremA, TremB and TremE. Two different exposition times were used for TremH: 20 h (boxed area) and 10 min.

PCR analysis performed with primers corresponding to the TIRs produced amplicons with sizes compatible with the Trem elements described above, confirming the results obtained in the genomic analysis (Figure 8). To confirm the PCR specificity, the amplicons were hybridized with probes containing internal regions of each Trem element (Figure 8). As expected, TremA was amplified in Pb03 and Pb18 (*P. brasiliensis*, phylogenetic species PS2 and S1, respectively) as well as in other isolates phylogenetically related to them, except for Penguin and Pb06 isolates. A similar amplification pattern was found for TremB in all PS2 and S1 isolates, except for isolate Pb14. TremA and TremB were not found in *Pb01-like *isolates. TremC was found in all isolates. A strong hybridizing 1.9 kb band was detected in the *Pb01-like *isolates. Two bands (1.9 and 1.8 kb) were amplified in the PS2 and S1 isolates; however, only the largest ones hybridized to the TremC probe. TremD was detected in all S1 isolates but not in the *Pb01-like *ones. TremE was amplified in *Pb01-like *isolates but not in S1 or PS2 isolates. A 2.3 kb TremF amplicon was detected in PS2 and S1 isolates. An additional hybridizing band of ~2.0 kb was detected in 7 S1 isolates, suggesting the presence of degenerate copies of TremF. In the *Pb01-like *isolates, a smaller PCR product was observed, but it did not hybridize with the TremF probe. The element TremH was amplified in all isolates we analyzed. Because of low copy-number of TremH in *Pb01-like *isolates, a longer exposure of the autoradiogram was needed to visualize the hybridizing bands. A strong hybridizing 2.0 kb band was seen in PS2 and S1 isolates. An additional hybridizing band of ~2.0 kb was detected in 9 S1 isolates, suggesting the presence of degenerate copies of TremH.

### Transcription of TremA and B elements

To isolate Trem transcripts, cDNAs from yeast forms were synthesized by RT-PCR using primers from TremA and TremB shown in Additional file 7. The RT-PCR products were separated by electrophoresis (Additional file 8), purified from the agarose gel and cloned into TA-vectors for sequencing. Transcripts of length corresponding to the unit size of the elements were amplified for Pb03 and Pb18 (*P. brasiliensis*, phylogenetic species PS2 and S1, respectively), but not for Pb01 (*P. lutzii*). Recombinant cDNA clones from isolate Pb03 were sequenced and found to be homologous to TremA (99.6% identity, NCBI accession number - FJ886812) and TremB (99.3% identity, NCBI accession number - FJ886813), confirming that these elements are transcribed. In conclusion, Trem elements are not silent in the genome.

## Discussion

We have carried out the first systematic search for DNA transposons in the genomic sequence of the dimorphic pathogenic fungus *Paracoccidioides*, isolates Pb01 (*P. lutzii*), Pb03 and Pb18 (*P. brasiliensis*, phylogenetic lineages PS2 and S1, respectively) (Assembly 1). The computational search strategy identified approximately 1300 putative transposon sequences (Table 1) in the genome of the 3 isolates, signaling the occurrence of class II TEs in the genus *Paracoccidioides*. We identified 8 families of transposons of the *Tc1/mariner *superfamily, based on the analysis of transposases and their associated TIRs and TSDs [20]. With the exception of TremG, in which the TIRs could not be defined, Trem elements are typical *Tc1/mariner *transposons exhibiting the TIR-transposase-TIR structure. *Tc1/mariner *superfamily is probably the most widely distributed transposon family in nature, being frequently found in the genomes of filamentous fungi [17,28]. However, there have to date been no descriptions of DNA transposons in dimorphic fungi. However, DNA transposons in the genus *Aspergillus *(subclass Eurotiomycetidae - NCBI, "taxonomy browser"), phylogenetically related to the dimorphic fungi, have already been identified [29,30].

Trem elements represent a significant proportion of the genome - around 1% of the genome of *P. brasiliensis *(isolates Pb03 and Pb18) and 0.6% of *P. lutzii *(Pb01) - indicating their successful proliferation in the genome. It is noteworthy that the majority (60-70%) of putative *Paracoccidioides *DNA transposons correspond to degenerated sequences (probably older insertions) that are difficult to classify; and were not included in this description. Among the remaining sequences, it may be possible to find elements belonging to the MITEs ("Miniature Inverted-repeat Transposable Elements"), a category of small, nonautonomous DNA transposons (100-500 bp) carrying preserved TIRs found in various organisms, including fungi [31].

Of the 464 Trem copies, 16 (3.5%) may be potentially autonomous active elements, as judged by the presence of intact TIRs and ORFs coding for transposases. Copies with truncated ORFs but preserved TIRs may be mobilized *in trans *by the enzyme coded by an intact copy [20]. HTH and DDE domains were preserved in the potentially active Trem elements. As previously observed in fungi, the glutamyl residue of the DDE domain was substituted by an aspartyl residue (DDD) [29,30]. The currently most known eukaryotic cut-and-paste transposons are DDE-superfamilies, *PiggyBac *and *Tc1/Mariner *being the only 2 superfamilies encoding DDD-transposases. Conservation of the triad signature (DDD) in *Tc1/mariner *transposons may reflect a catalytic mechanism similar to that described for the DDE motif, which probably coordinate divalent metal ions to promote catalysis of DNA cleavage and ligation.

The CENPB domain was found in TremA, TremB and TremF elements. This domain has been identified in mammalian centromeric proteins and eukaryotic transposases, but not in any archaeal, bacterial or plant proteins. It may be implicated in the recognition of the terminal inverted repeats of transposons. The functional similarity between CENPB domains could be the result of domestication of transposons by the host genome to act as its own genes or regulatory elements [29,32-34]. The N-terminal HTH domain, also found in the truncated elements TremC and TremE, has similarity with the "pipsqueak" DNA-binding domain of *Drosophila*, a family of eukaryotic proteins that recognize and bind to DNA sequences [35]. The terminal inverted repeats were identified in 7 of the 8 Trem elements. The inverted regions had sizes compatible with those of other elements belonging to the *Tc1/mariner *superfamily described in fungi, and also had a 17 bp internal direct repeat, as previously reported [17,29,36]. It was suggested that this direct repetition could be the recognition and binding of the HTH and CENPB transposase domains [37].

An interesting finding was the presence of conserved Cx2Cx4Hx4C (CCHC) motifs indicating zinc finger (ZF) domains in the C-terminal ends of transposases of at least 3 Trem elements (TremA, TremB and TremD). The ZF, which is present in a wide range of proteins, forms a structure stabilized by co-ordination of a divalent zinc cation [Zn (II)], with cysteine or histidine residues as ligands. ZF are structurally diverse and present among proteins with a broad range of cellular functions, such as replication and repair, transcription and translation, metabolism and signaling, cell proliferation and apoptosis. Krishna et al. [38], who described 8 fold groups of ZF, discussed their similarities and differences with functional implications. The fold group 1 consists of 2 families: C2H2 fingers and IAP (inhibitor of apoptosis) domains. IAP contains a CCHC pattern that coordinates a zinc ion, a pattern similar to that found at the C-terminal region of Trem elements transposases. The ZF is responsible for binding to DNA or RNA in some proteins, or for the protein-protein interaction in others. Ohta et al. [39] described transposases encoded by transposable elements of the Insertion Sequences 1 (IS1 family) of *Escherichia coli *as having N-terminal regions containing a C2C2 ZF motif. They showed that the ZF motif, as well as the HTH motif, is essential for transposition promoted by IS1transposase because of their involvement in binding specifically to TIRs. IS1 transposases with an amino acid substitution in the HTH or ZF motif lose the ability to promote transposition, indicating that 2 domains are responsible for TIR-specific DNA binding in promoting transposition. It is likely that the 2-domain structure is required for binding to TIRs, not only in IS1transposase, but also in transposases encoded by some other transposable elements, in order to place a catalytic domain of transposases in a region near the TIR end, where strand transfer reactions occur in transpososomes. These observations suggest the possibility that transposases encoded by some elements have 2 domains involved in the TIR-specific DNA-binding. In eukaryotes, a *Drosophila *transposable element P encodes a transposition inhibitor that has a CCHC region forming a ZF that plays an important role in the binding to the TIR sequences at the termini of the P element [38,39].

When transposons move along the genome, footprints known as TSDs ("Target Site Duplications") can be formed that flank the element. Most transposon superfamilies may have a characteristic TSD with a specific size and sequence [20]. TSDs were found in 6 out of the 8 Trem elements. It was not possible to determine the TIRs in TremG and consequently find the flanking TSD. The copies of TremH are truncated and probably inactivated, resulting in loss or alteration of the TSD, consequently preventing its identification.

As the DNA transposons of the *Tc1/mariner *superfamily have the dinucleotide TA as their insertion site, they can be inserted within virtually any genomic region. However, regional preferences have been observed for many elements which are inserted in specific regions of the genome [40]. The comparison of the number and identity of nested insertions within individual transposons has been used to estimate the relative ages of transposons. It is reasonable to assume that the older elements are subjected to interruptions by more recently active elements. Older elements should therefore have higher proportions of nested insertions than younger elements. Assuming that DNA transposon insertions occur randomly throughout the genome, there should be a higher proportion of nested insertions for older Trem elements than for younger ones. The following evidence could support the hypothesis of recent transposition and expansion of Trem elements in the genome: (a) the absence of nested Trem copies inside other transposons; (b) the presence of intact open reading frames; and (c) the presence of highly similar copies in some Trem elements. Analysis of Southern blots and chromoblots of genomic DNA from isolates from 2 different species, *P. lutzii *(Pb01) and *P. brasiliensis *(Pb03 and Pb18), corroborates the *in silico *data in relation to the presence and distribution of Trem elements in these genomes.

Trem elements do not appear to have been acquired simultaneously by the *Paracoccidioides *sequenced genomes, as indicated by finding of different copy-numbers among Trem families. The elements TremC and TremH are present in a large number of copies, most of which are truncated elements with stop codons. Unlike TremC and TremH, TremD is only present in 5 copies, 4 of which have integral ORFs, just in isolate Pb18 (*P. brasiliensis*, phylogenetic species S1). This pattern is compatible with a recent insertion in the genome, as TremD would not yet have been inactivated by the genome defense system.

Several recent studies have proposed the existence of different phylogenetic species in the taxon *P. brasiliensis*. Matute et al. [10] analyzed the genetic structure of 65 *P. brasiliensis *isolates, concluding that they can be grouped into 3 distinct phylogenetic species: S1 (including isolate Pb18), PS2 (including isolate Pb03) and PS3 (composed exclusively of Colombian isolates). In a study of 21 *P. brasiliensis *isolates, 14 of which had been included in the above study, Carrero et al. [11] reached a similar conclusion for all the isolates except isolate Pb01, which they suggested was a new phylogenetic species in the genus *Paracoccidioides*. Recently, Teixeira et al. [12], analyzing 88 isolates of the fungus, found that 17 were genotypically similar, belonging to the *Pb01-like *group. They estimated that the S1/PS2/PS3 species clade and the *Pb01-like *new species, for which the name *P. lutzii *was proposed, shared a common ancestor approximately 32 million years ago.

We used PCR to analyze the distribution of Trem elements in 17 clinical isolates and one environmental isolate formerly known as *P. brasiliensis*. Previous reports using RAPD for typing *P. brasiliensis *indicated that the use of certain primers and conditions allowed the discrimination of isolates with very low genetic similarity clustering the most similar ones. The primers chosen discriminated of *P. brasiliensis *isolates into groups with very low genetic similarity indexes (17-35% when the Pb01 isolate was included or at least 50-60% when it was not) [5,26,41]. Probably for this reason our RAPD analysis resulted in the clustering of isolates previously known to belong to a certain phylogenetic species (*Pb01-like*, S1 and/or PS2), which could be a reference for the characterization of those of unknown genetic relatedness (Table 3, see isolates Ed01, 1578 and utero). The 18 isolates consisted of the sequenced Pb01 and 2 *Pb01-li*ke isolates (*P. lutzii*) [12]; 3 isolates previously classified as PS2 (including isolate Pb03); and 12 isolates identified as S1 (including Pb18). PS3, the fourth known phylogenetic species reported to be exclusively in Colombia was not represented [10]. The isolates were grouped in accordance with Matute et al.[10], Carrero et al. [11], Teixeira *et al*. [12] and the results of RAPD analysis (Figure 7) [27]. Isolates Penguin and Pb12 are known to be *P. brasiliensis *(S1) but do not cluster with the other S1 isolates (Figure 7). These isolates come from different geographic regions, Uruguay and Argentina, while the majority is Brazilian (13 isolates, Table 3). It is interesting that a fungal associated feature that has been reported to correlate by RAPD branching or clustering of *Paracoccidioides *refers to the isolate's geographic origin [13]. The non-grouping of all S1 isolates would be explained by the fact that multilocus sequencing studies employ nuclear genes while RAPD would arbitrarily reveal polymorphic regions along the genomes, including intergenic and/or repetitive sequence.

The DNA transposons TremC and TremH were identified in all the isolates tested, indicating that these elements would have already been present in a hypothetical common ancestor rather than have been acquired horizontally after the 3 phylogenetic species diverged from one another. In contrast, TremE was only found in Pb01 and *Pb01-like *isolates, and TremA, TremB, and TremF were found in the S1 and PS2 isolates, while the element TremD was almost exclusively found in S1 isolates, suggesting that these DNA transposons could have been acquired horizontally after separation of the 3 phylogenetic species. Although TremD and TremE share approx 70% similarity at the amino acid level, they were found exclusively in the S1 and *Pb01-like *isolates, respectively, which are phylogenetically distant. This suggests that these elements would have been acquired by horizontal transfer after the split between S1/PS2 (*P. brasiliensis*) and *Pb01-like *species (*P. lutzii*). This hypothesis is supported by the finding of hypothetical protein sequences of *A. capsulatum *in GenBank (accession numbers XM 001537507 and XM 001543155) that yielded an e-value of 0.0 for the elements TremD and TremE, respectively (i.e. their sequences are identical or have at least 80% identity). However, the absence of TremD and TremE could also be explained by the loss of these elements in some strains.

Transcription of TremA and TremB suggests that they could be active elements. When transposing, the elements generate double-strand DNA breaks, which are repaired by the cellular machinery [42]. Hence, in addition to the direct effects of transposition, such as gene inactivation, recombination events promoted by repair of the double strand would also take place. Transposition of these elements could be a factor involved in the genetic variability observed in the *P. brasiliensis *species complex. The identification of active elements would serve as a basis for the development of mutational tools, as transposons could be efficient vectors for modifications.

## Conclusions

The strategy developed in the present work proved effective in identifying and characterizing DNA transposons in the newly generated sequence assemblies of isolates of 3 phylogenetic species of the *P. brasiliensis *complex. The most intact insertions allowed the classification of 7 new families and a new subfamily of elements (TremA - H) that share identity with the *Tc1/mariner *superfamily, distinct lineages not yet identified in dimorphic fungi. Three full-length, potentially functional autonomous elements were characterized (Trem A, B and D). Further studies to show their active transposition would be interesting. The phylogenetic analysis supports the hypothesis that Trem families derived from a single ancestral sequence and split into 2 lineages (TremA - F; TremG - H). The occurrence of Trem families is unequal in the genomic sequence of the isolates Pb01, Pb03 and Pb18 and experimental evidence from the other 15 typed isolates indicated that some Trem families (TremC and H) were shared while other Trem families were harbored exclusively by *Pb01-like *species (*P. lutzii*) (TremE) or S1/PS2 species (*P. brasiliensis*) (TremA, B, D, and F). It would be interesting to verify the occurrence of distinct Trem families in a larger number of *P. lutzii *and *P. brasiliensis *isolates from different origins (either clinical or environmental), and from different geographic regions, also including the phylogenetic species PS3, not represented here, in such a way as to expand and explore the potential of these newly presented genetic agents as sources of phylogenetic and epidemiological knowledge.

## Methods

### Bioinformatic analysis

A retrieval based on specific keywords (transposase, transposons, transposable) was performed against the *P. brasiliensis *Functional and Differential Genome Project EST annotated database (PbDBEST) http://www.biomol.unb.br/[15,24] to identify sequences containing particular annotation terms related to transposable elements. At the time of writing, 2 contigs have been identified: Contigs 530 (673 bp) and 1938 (637 bp), GenBank accession numbers CN240498 and CN247880, respectively.

These contigs were further used in similarity searches using BLAST algorithm [38] (specifically blastn and tblastx with default parameters) against the first draft of the genomic sequence of *P. brasiliensis *isolates Pb03, Pb01 and Pb18 released by the Fungal Genome Initiative Project (FGI) of The Broad Institute http://www.broad.mit.edu.

### Databases

The *P. brasiliensis *genomic sequences of isolates Pb01, Pb03 and Pb18 from the Fungal Genome Initiative Project (FGI) of The Broad Institute were downloaded (*Paracoccidioides brasiliensis *Database) http://www.broad.mit.edu/annotation/genome/paracoccidioides_brasiliensis/Downloads.html and used in the *in silico *analysis described in this work. The locally compiled database of *P. brasiliensis *sequences, built by parsing sequences from the original database, was used for sequence similarity searches, using the BLAST [43] algorithm.

### Transposon mapping and TIR identification

To identify and map transposable elements in the genomic sequences of *P. brasiliensis *isolates Pb03, Pb01 and Pb18, an approach was developed that we called "Class-Specific Method for transposable element identification". This method takes into account the advantage of the knowledge of particular genomic features characteristic of specific classes of the elements to aid their identification.

The homology-based method we developed identifies transposable elements by comparing genomic query sequences (Pb01, Pb03 and Pb18) against themselves. Sequence comparison by BLAST algorithm took into account query hits identified in a search using only the top strand (-S 1 parameter) to search against database, a word size of 7. To prevent long high-scoring segment pairs (HSPs) being broken up into smaller ones because of low-complexity segments and repeats, the default filter (DUST) was turned off. Specific databases containing each genomic sequence of *P. brasiliensis *isolates were built converting the DNA sequence into its reverse-complement counterpart. Similarity results that emerge from this self comparison were parsed by 2 in-house developed PERL (Practical Extraction and Report Language) scripts. These scripts specifically convert the standard BLAST output into a tabular format and allow the identification of pairs of inverted repeats at approximately 2.5 Kb from each other (i.e. the average distance between TIRs of *Tc1/mariner *elements).

The sequence stretches containing flanking inverted repeats identified using this initial criteria were finally compared in terms of the putative gene products encoded against NCBI nr protein database to identify putative transposable elements.

### Annotation

The annotation and graphical output of the supercontigs were performed using ARTEMIS [44] software http://www.sanger.ac.uk/Software/Artemis/ and 4 in-house developed PERL scripts to analyze and format the results.

Classification of the DNA transposons follows the nomenclature previously established, and families and subfamilies were disclosed according to the standard principles of TE classification [20,45-47]. In this report we define the DNA transposon family by DNA sequence as described by Wicker at al. [20]: 2 elements belong to the same family if they share 80% (or more) sequence identity in at least 80% of their coding region or internal domain or within their terminal repeats (or both). All consensus sequences (Additional file 9) were submitted to Repbase using the Repeat Masking algorithm to identify related elements http://www.girinst.org/censor/index.php.

### Multiple sequence alignment and phylogenetic inference

Global multiple sequence alignments using amino acid sequences from identified transposases and nucleotide sequences from identified transposons were performed using the ClustalX algorithm [48], followed by visual inspection and manual adjustment with SeaView [49]http://pbil.univ-lyon1.fr/software/seaview.html and GeneDoc http://www.psc.edu/biomed/genedoc/.

Phylogenetic reconstruction of the sequences aligned by ClustalW [48] were performed using Neighbor-joining method implemented in MEGA 4 [50]. Trees were constructed using the equal-input model with 5000 bootstrap replicates. Branch lengths reflect the sequence divergence (see the scale bar). The amino acid sequences used in the phylogenetic reconstruction are available at: http://www.girinst.org/repbase/update/browse.php with the follow locus names: Mariner-6_AN, Mariner-3_AF, Mariner-7_AN, Mariner-1_AF, Mariner2_AO, Mariner3_AO, Mariner-5_AF, Mariner-7_AF, Mariner-3a_AN, Mariner-3_AN, Mariner-1_AN, Mariner-4_AN, Mariner-5_AN, Mariner5_AO. The amino acid sequences from pogo_ DM and Pot2_MG (NCBI accession number S20478 and XP364943, respectively) were also used.

### *P. brasiliensis *isolates and growth conditions

The characteristic *P. brasiliensis *isolates are listed in Table 3. Fungal isolates were maintained by periodic subculturing in PYG medium (yeast extract 5 g, bacto-peptone 10 g, dextrose 15 g, and Agar 15 gl^-1^, pH 6.3) at 35-37°C [8].

### DNA and RNA extraction

Total DNA was extracted from the yeast culture following previously described protocols involving maceration of frozen cells, with minor modifications [51]. Total RNA was extracted from the yeast cultures as previously reported [52].

### PCR

PCR reactions were carried out on 10 ng of DNA in a 50 μL reaction mixture containing 10 mM Tris-HCl, pH 9, 1.5 mM MgCl_2_, 50 mM KCl, 100 mM of each dNTP, 100 pmol of each oligonucleotide and 1 unit of Taq DNA Polymerase (Promega). Each PCR was carried out in a programmable temperature controller (Eppendorf) for 25 cycles. The cycling conditions were as follows: denaturing at 94°C for 1 min, annealing at the Tm for each primer for 30 s, and extension at 72°C for 1 min. At the end of the 25^th ^cycle, the heat-denaturing step was omitted and extension was allowed to proceed at 72°C for 5 min. The primers and respective Trem are listed in Additional file 7.

### RAPD analysis

RAPD analysis was performed as previously described [5,26,41], using the primers OPG03 (GAGCCCTCCA), OPG11 (TGCCCGTCGT), OPG15 (ACTGGGACTC), OPG16 (AGCGTCCTCC), OPG18 (GGCTCATGTG) and OPO06 (CCACGGGAAG).

PCR reactions were carried out in 10 mM Tris-HCl, pH 9, 2 mM MgCl_2_, 50 mM KCl, 200 mM of each dNTP, and 0.25 mM of each oligonucleotide in a 20 μL reaction mixture containing 1 unit of Taq DNA Polymerase (Phoneutria). The cycling conditions were as follows: denaturing at 94°C for 3 min followed by 40 cycles of denaturing at 94°C for 45 s, annealing at 36°C for 45 s and extension at 72°C for 105 s. At the end of the 40^th ^cycle, the heat-denaturing step was omitted and extension was allowed to proceed at 72°C for 3 min. The products were separated by polyacrylamide gel electrophoresis and silver stained (5% silver stain). Digitized gel images were analyzed by LabImage version 2.7.1, and dendrograms were generated by TFPGA software version 1.3.

### Transcriptional analysis

First- and second-strand cDNAs were prepared using SuperScript One-Step reverse transcriptase PCR (RT-PCR) with Platinum *Taq *according to the manufacturer's instructions (Life Technologies). Specific forward or reverse primers based on the nucleotide sequences of the elements TremA and TremB were used with oligo(dT) to amplify sequences by PCR. Genomic DNA false positive results were excluded by performing the reaction in the absence of reverse transcriptase, and with a treatment of RNA samples, prior to the RT-PCR reaction, with DNase I, RNase free.

### Cloning and sequencing

TremA and TremB genomic and cDNA amplicons were then cloned into plasmid pGEM-T Easy vector (Promega) and transformed into *Escherichia coli *DH5 *α *competent cells. Nucleotide sequences of cDNA clones were determined by the dideoxynucleotide chain termination method, using BigDye Terminator cycle sequencing chemistry (Applied Biosystems) in an ABI PRISM 377 DNA sequencer.

### Blotting analysis

For Southern blot analysis, PCR amplifications or DNA samples digested with the restriction enzyme *Bgl*II, were separated by electrophoresis on 0.8% agarose gels and stained with ethidium bromide (0.5 μg/mL). For chromoblot analysis, the separation of chromosome-sized *P. brasiliensis *DNA molecules by PFGE (pulsed field gel electrophoresis) was performed as described by Feitosa et al. [8]. The agarose gels were incubated with 0.25 M HCl for 30 min, denatured with 0.5 M NaOH/1 M NaCl for 30 min, neutralized with 1 M Tris-base/0.5 M NaCl for 20 min and transferred onto nylon membranes in 20 × SSC (0.15 M NaCl and 0.015 M sodium citrate). The membranes were prehybridized in a solution containing 50% formamide-5×, 5 × SSC, 0.5% Denhardt's solution, 0.1 mg/mL salmon sperm DNA and 0.1 mg/mL tRNA at 42°C for 1 h, and then hybridized overnight at the same temperature with a ^32^P-labeled probe. The probes used in the hybridization experiments were derived from the internal region of each element, including the ORF. Following hybridization, the membranes were washed 3 times (30 min each) in 2 × SSC containing 0.1% SDS at 42°C, 1 × SSC containing 0.1% SDS and 0.1 × SSC containing 0.1% SDS at 56°C, before being exposed to X-ray film.

## Abbreviations

RAPD: random amplification of polymorphic DNA; RFLP: restriction fragment length polymorphism; GCPRS: genealogic concordance phylogenetic species recognition; TE: transposable element; TIR: terminal inverted repeat; HTH: Helix-Turn-Helix_pipsqueak; TSD: target site duplications; MITE: miniature inverted-repeat transposable element; FGI: Fungal genome initiative; GIRI: genetic information research institute; Trem: transposable element mariner; ZF: zinc finger; HSPs: high-scoring segment pairs; DUST: default filter; PERL: practical extraction and report language

## Authors' contributions

MMM participated in the experimental design, bioinformatics analysis and annotation, experiments on physical mapping and transcriptional analysis, and manuscript preparation. TZ, PCS, RRMB and ACPG participated in and supported experiments in physical mapping, cloning and sequencing, transcriptional and RAPD analysis. MSSF, MB, CMSA and RP were the principal investigators for the transcriptome and genome projects of *P. brasiliensis *and also participated in manuscript preparation. JCR designed and performed the bioinformatics analysis. JFS and PSC designed, coordinated, supervised and participated in the interpretation, discussion of results and manuscript preparation. All authors read and approved the final manuscript.

## Supplementary Material

Additional file 1 **Results of Trem analysis performed with the Repeat Masking script using the Girinst database**. The consensus sequence of Trem elements were submitted to Repbase using Repeat Masking algorithm to identify related elements in Girinst database http://www.girinst.org/repbase. Click here for file

Additional file 2 **List of all Trem insertions in the genome of *P. brasiliensis *isolates Pb01, Pb03 and Pb18**. The complete list of all Trem insertions, indicating the position of each element, the presence or absence of the TIRs and TSD, in the genome sequence of isolates Pb01, Pb03 and Pb18. Click here for file

Additional file 3 **Distribution of Trem elements in the genome**. Information of each supercontig: GenBank accession number, size and number of Trem elements. In the column ORF are presented only the insertions of Trem elements that show complete, non truncated ORFs. Click here for file

Additional file 4 **Supercontig view showing the distribution of Trem elements in the genome of *P. brasiliensis *isolate Pb03**. Each supercontig is represented by a dashed line in scale. Below each dashed line the Trem insertions along the supercontig are represented. The GenBank accession number of contigs is indicated in parentheses. The coordinates of each Trem element (genome location, length, TIR) displayed in the supercontig view are accessible in Additional file 1. Click here for file

Additional file 5 **Supercontig view showing the distribution of Trem elements in the genome of *P. brasiliensis *isolate Pb18**. Each supercontig is represented by a dashed line in scale. Below each dashed line the Trem insertions along the supercontig are represented. The GenBank accession number of contigs is indicated in parentheses. The coordinates of each Trem element (genome location, length, TIR) displayed in the supercontig view are accessible in Additional file 1. Click here for file

Additional file 6 **Supercontig view showing the distribution of Trem elements in the genome of *P. brasiliensis *isolate Pb01**. Each supercontig is represented by a dashed line in scale. Below each dashed line the Trem insertions along the supercontig are represented. The GenBank accession number of contigs is indicated in parentheses. The coordinates of each Trem element (genome location, length, TIR) displayed in the supercontig view are accessible in Additional file 2 Click here for file

Additional file 7 **Sequences of primers used to amplify Trem elements by PCR and RT-PCR**. Table with the sequence of the primers used to amplify Trem elements by PCR and RT-PCR. Click here for file

Additional file 8 **Transcription of TremA and TremB elements in *P. brasiliensis *isolates**. RT-PCR was carried out on total RNA from the yeast form of isolates Pb01, Pb03 and Pb18 using specific primers for the ORFs of TremA and TremB (see Additional file 7). Click here for file

Additional file 9 **Consensus sequences of Trem element**. The nucleotide consensus sequence of Trem elements identified. Click here for file
