# Supplementary material for: Identification and characterization of Tc1/mariner-like DNA transposons in genomes of the pathogenic fungi of the Paracoccidioides species complex
Source: BMC Genomics. 2010 Feb 23;11:130. doi: 10.1186/1471-2164-11-130 (PMC2836289; doi:10.1186/1471-2164-11-130)
Supplement: Additional file 1 — Results of Trem analysis performed with the Repeat Masking script using the Girinst database. The consensus sequence of Trem elements were submitted to Repbase using Repeat Masking algorithm to identify related elements in Girinst database http://www.girinst.org/repbase. [file 1471-2164-11-130-S1.DOC]

**Additional file 1 – Results of Trem analysis performed with the Repeat Masking script using the Girinst database.**

The consensus sequence of Trem elements were submitted Repbase using Repeat Masking algorithm to identify related elements in Girinst database (<http://www.girinst.org/repbase>).

| AF - *Aspergillus fumigatus* |
| --- |
| AN - *Aspergillus nidulans* |
| AO - *Aspergillus oryzae* |
