# Supplementary figures and images for: Identification and characterization of Tc1/mariner-like DNA transposons in genomes of the pathogenic fungi of the Paracoccidioides species complex
Source: BMC Genomics. 2010 Feb 23;11:130. doi: 10.1186/1471-2164-11-130 (PMC2836289; doi:10.1186/1471-2164-11-130)

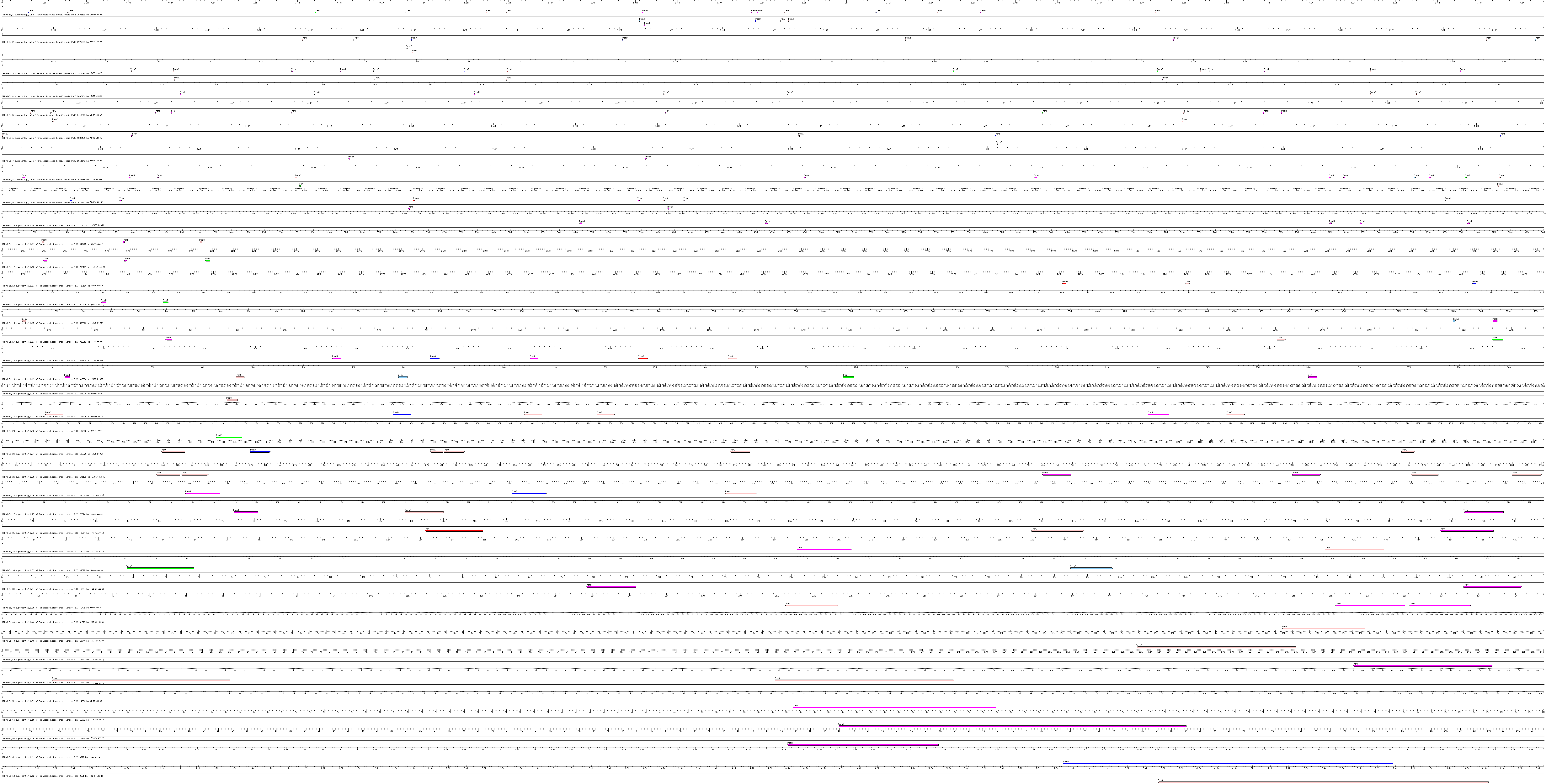

Supplement: Additional file 4 — Supercontig view showing the distribution of Trem elements in the genome of P. brasiliensis isolate Pb03. Each supercontig is represented by a dashed line in scale. Below each dashed line the Trem insertions along the supercontig are represented. The GenBank accession number of contigs is indicated in parentheses. The coordinates of each Trem element (genome location, length, TIR) displayed in the supercontig view are accessible in Additional file 1. [file 1471-2164-11-130-S4.PNG]

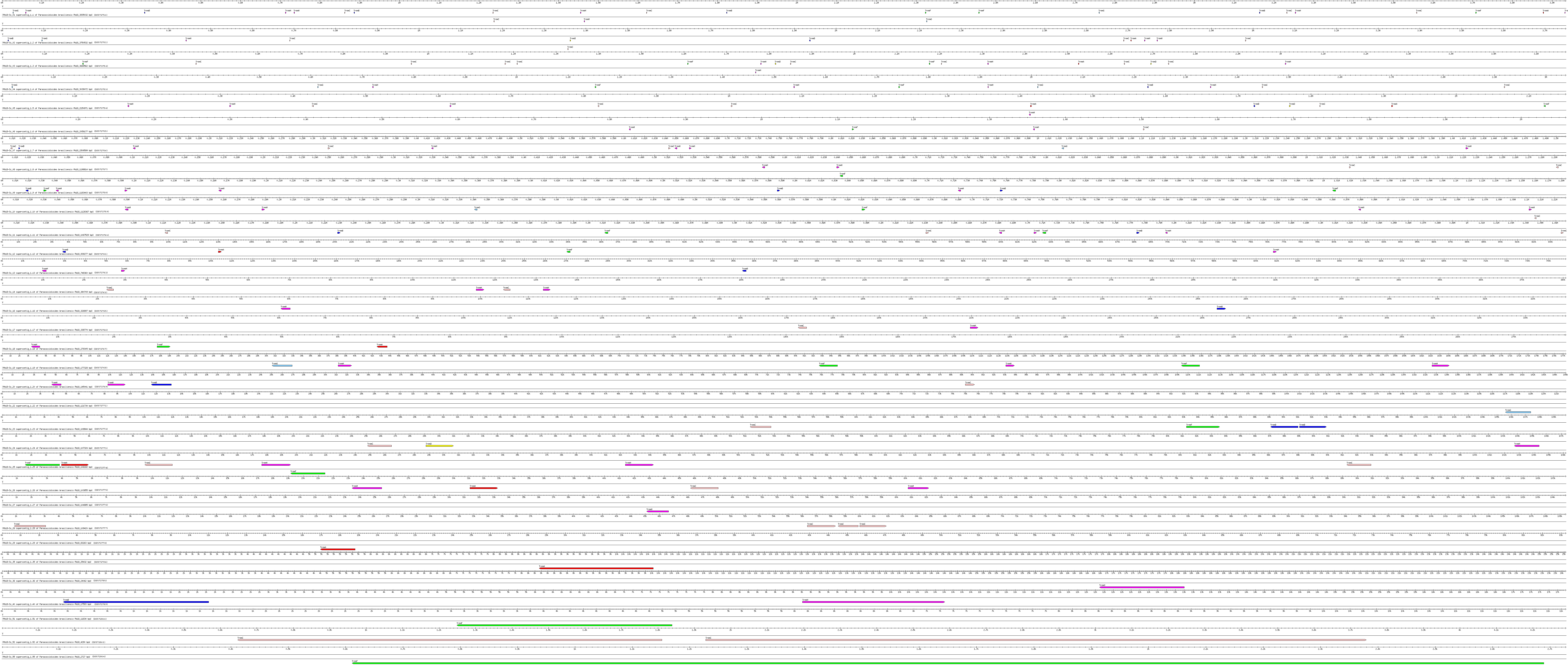

Supplement: Additional file 5 — Supercontig view showing the distribution of Trem elements in the genome of P. brasiliensis isolate Pb18. Each supercontig is represented by a dashed line in scale. Below each dashed line the Trem insertions along the supercontig are represented. The GenBank accession number of contigs is indicated in parentheses. The coordinates of each Trem element (genome location, length, TIR) displayed in the supercontig view are accessible in Additional file 1. [file 1471-2164-11-130-S5.PNG]

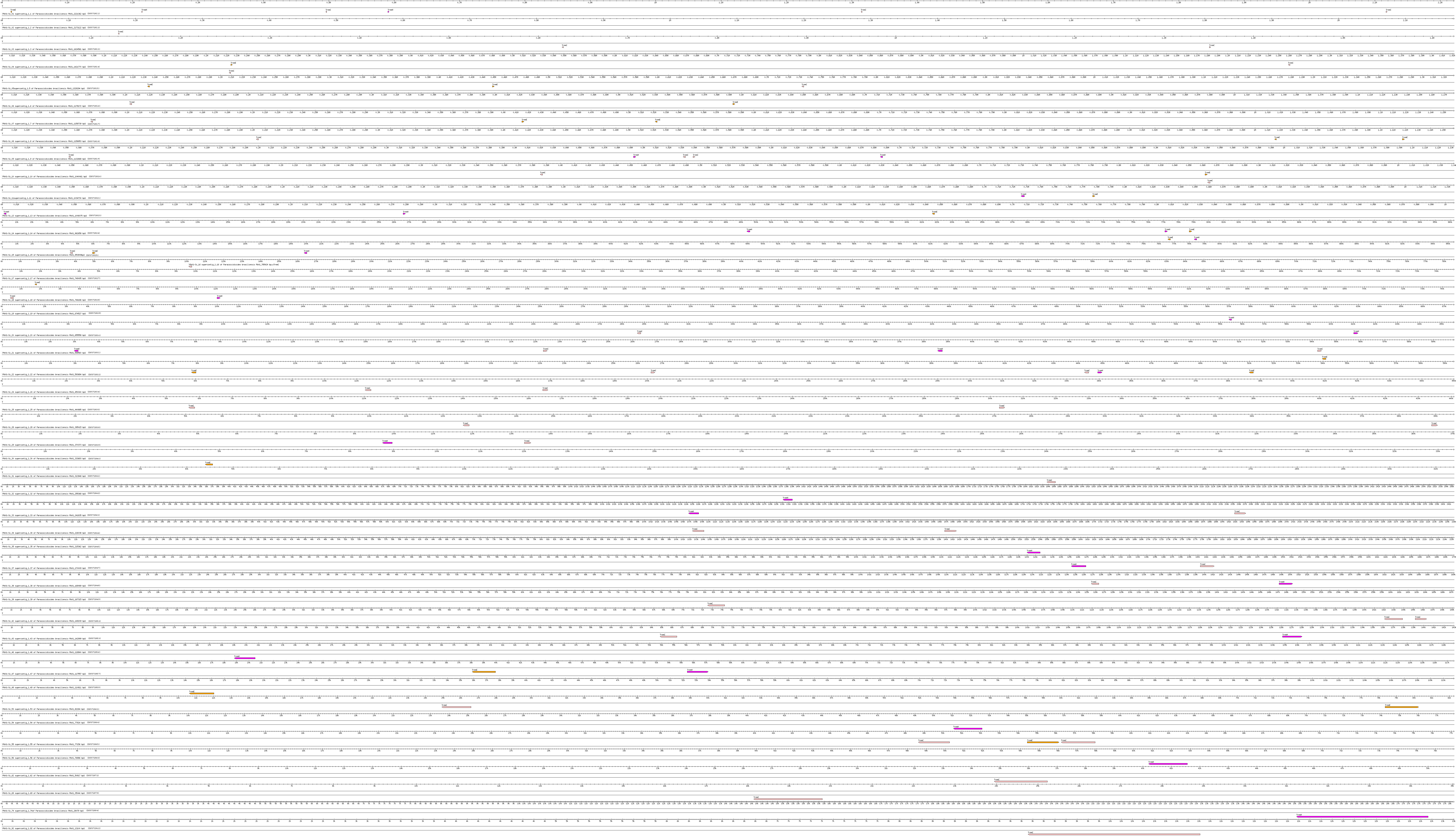

Supplement: Additional file 6 — Supercontig view showing the distribution of Trem elements in the genome of P. brasiliensis isolate Pb01. Each supercontig is represented by a dashed line in scale. Below each dashed line the Trem insertions along the supercontig are represented. The GenBank accession number of contigs is indicated in parentheses. The coordinates of each Trem element (genome location, length, TIR) displayed in the supercontig view are accessible in Additional file 2 [file 1471-2164-11-130-S6.PNG]

## Slide 1
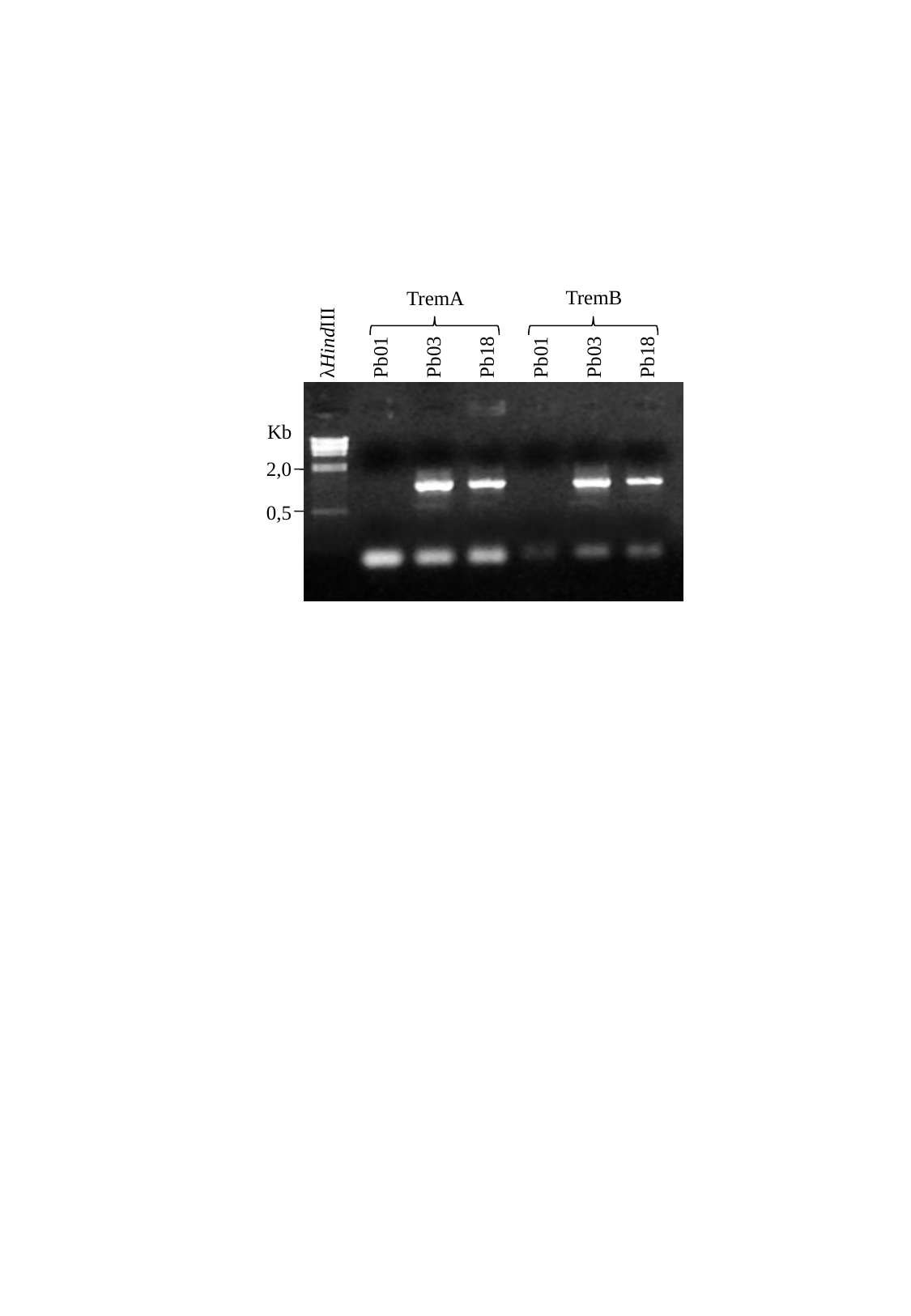

TremB
TremA
λHindIII
Pb01
Pb03
Pb18
Pb01
Pb03
Pb18
Kb
2,0
0,5

Supplement: Additional file 8 — Transcription of TremA and TremB elements in P. brasiliensis isolates. RT-PCR was carried out on total RNA from the yeast form of isolates Pb01, Pb03 and Pb18 using specific primers for the ORFs of TremA and TremB (see Additional file 7). [file 1471-2164-11-130-S8.PPT]
