# Supplementary material for: Identification and characterization of Tc1/mariner-like DNA transposons in genomes of the pathogenic fungi of the Paracoccidioides species complex
Source: BMC Genomics. 2010 Feb 23;11:130. doi: 10.1186/1471-2164-11-130 (PMC2836289; doi:10.1186/1471-2164-11-130)
Supplement: Additional file 7 — Sequences of primers used to amplify Trem elements by PCR and RT-PCR. Table with the sequence of the primers used to amplify Trem elements by PCR and RT-PCR. [file 1471-2164-11-130-S7.DOC]

**Additional file 7 – Sequences of primers used to amplify Trem elements by PCR and RT-PCR.**

| **Primer** | **Annealing temp (°C)** | **Oligonucleotide sequence (5’🡪3’)** |
| --- | --- | --- |
| 530 Fw | 58 | CTCATCCATCTCAACGCGCAAT |
| 530 Rrev | 60 | TCTGGCGGAATCCCATGCTC |
| 1938 Fw | 54 | GCCACCAATTCGCAATAAGA |
| 1938 Rev | 53 | CATTCACGATTTCCAGGTTGT |
| TIR TremA | 55 | TAGTCCGACACCTAACCGATC |
| TremA ORF Fw | 65 | TCAGCTCCATGACTTCCTTGGT |
| TremA ORF Rev | 64 | CCACGTACACAAATGCTCAACT |
| TIR TremB | 55 | TAACCAATCACTCAACTGGCC |
| TremB ORF Fw | 62 | gtaaagccttgttctcttgagc |
| TremB ORF Rev | 60 | GAAATCCCAGTCTCAACGCCA |
| TIR TremC | 52 | ACGTAATCAATAAGCGAGTCG |
| TremC int Fw | 58 | CTCTACATGCCACCACATT |
| TremC int Rev | 57 | ATTCAAGTCTTCTTCAATCCTC |
| TIR TremD | 65 | TCCACAAGCGAGCGGGCCA |
| TremD int Fw | 58 | GTGATTATGCCACCAATTCG |
| TremD int Rev | 57 | TTATAGTACAATAGGACTAGGG |
| TIR TremE | 53 | GTAATTCCACACCGAGTCG |
| TremE int Fw | 60 | CGCGCAATTATGCCACCAA |
| TremE int Rev | 59 | TCCGTACACTCACTTTAAAACTG |
| TIR TremF | 59 | TAAACCGCCACACAACGCAC |
| TremF int Fw | 58 | ATGGGCGTCATGCAAATCA |
| TremF int Rev | 56 | TAGTTAATCCAAAAATCTAGATGG |
| TIR TremH | 52 | CCACACCAAAACGATAAAAACA |
| TremH int Fw | 57 | CACCACAATGTCAAATAAGGATA |
| TremH int Rev | 60 | CGTCACTCCTTATTCTGCTC |
